# The Antibiotic Neomycin Enhances Coxsackievirus Plaque Formation

**DOI:** 10.1128/mSphere.00632-18

**Published:** 2019-02-20

**Authors:** Mikal A. Woods Acevedo, Andrea K. Erickson, Julie K. Pfeiffer

**Affiliations:** a Department of Microbiology, University of Texas Southwestern Medical Center, Dallas, Texas, USA; University of Zurich

**Keywords:** antibiotics, coxsackievirus, microbiota, neomycin, reovirus

## Abstract

Coxsackievirus typically infects humans via the gastrointestinal tract, which has a large number of microorganisms collectively referred to as the microbiota. To study how the intestinal microbiota influences enteric virus infection, several groups have used an antibiotic regimen in mice to deplete bacteria. These studies have shown that bacteria promote infection with several enteric viruses. However, very little is known about whether antibiotics influence viruses in a microbiota-independent manner. In this study, we sought to determine the effects of antibiotics on coxsackievirus B3 (CVB3) using an *in vitro* cell culture model in the absence of bacteria. We determined that an aminoglycoside antibiotic, neomycin, enhanced the plaque size of CVB3 strain Nancy. Neomycin treatment did not alter viral attachment, translation, or replication. However, we found that the positive charge of neomycin and other positively charged compounds enhanced viral diffusion by overcoming the negative inhibitory effect of sulfated polysaccharides present in agar overlays. Neomycin and the positively charged compound protamine also enhanced plaque formation of reovirus. Overall, these data provide further evidence that antibiotics can play noncanonical roles in viral infections and that this should be considered when studying enteric virus-microbiota interactions.

**IMPORTANCE** Coxsackieviruses primarily infect the gastrointestinal tract of humans, but they can disseminate systemically and cause severe disease. Using antibiotic treatment regimens to deplete intestinal microbes in mice, several groups have shown the bacteria promote infection with a variety of enteric viruses. However, it is possible that antibiotics have microbiota-independent effects on viruses. Here we show that an aminoglycoside antibiotic, neomycin, can influence quantification of coxsackievirus in cultured cells in the absence of bacteria.

## Abstract (precis)

Coxsackieviruses primarily infect the gastrointestinal tract of humans, but they can disseminate systemically and cause severe disease. Using antibiotic treatment regimens to deplete intestinal microbes in mice, several groups have shown the bacteria promote infection with a variety of enteric viruses. However, it is possible that antibiotics have microbiota-independent effects on viruses. Here we show that an aminoglycoside antibiotic, neomycin, can influence quantification of coxsackievirus in cultured cells in the absence of bacteria.

## INTRODUCTION

Coxsackievirus B3 (CVB3) is a cardiotropic nonenveloped RNA virus belonging to the *Enterovirus* genus of the *Picornaviridae* family. CVB3 is an important human pathogen which can cause a wide range of diseases, including myocarditis, cardiac arrhythmias, aseptic meningitis, type 1 diabetes, gastrointestinal distress, and death (1–5). CVB3 has been implicated in more than 40,000 infections a year in the United States alone, and there are no current treatments or vaccines for CVB3 infections (6).

Within the gastrointestinal tract resides a microbial ecosystem of approximately 10^14^ organisms, which play a crucial role in host homeostasis (7). The intestinal microbiota can also influence infection with orally acquired enteric viruses (8–10). Alterations in microbiota, for example, through antibiotic treatment, can influence enteric pathogen susceptibility (8–10). However, not much is known about direct effects of antibiotics on enteric viruses.

Antibiotics can have a variety of microbiota-independent effects on mammalian cells. Antibiotics can elicit profound changes in host gene expression in both conventional and germfree mice (11), alter mammalian metabolic pathways and impair the phagocytic activity of immune cells (12), induce mitochondrial dysfunction (13, 14), and inhibit histone demethylases (15). Additionally, Gopinath et al. recently demonstrated that aminoglycoside antibiotics can confer microbiota-independent antiviral resistance against both DNA and RNA viruses by upregulating expression of interferon-stimulated genes (16).

In this study, we examined the effect of antibiotic treatment on CVB3 infection of cultured cells in the absence of bacteria. From a group of antibiotics that is commonly given to mice in microbiota depletion studies, we found that neomycin increases the plaque size of CVB3. Notably, treatment with neomycin did not have an apparent effect on viral replication in single-cycle growth curves. We determined that plaque size enhancement by neomycin was most likely due to its positive charge overcoming the inhibitory negative charge of agar overlays, thus aiding viral diffusion.

## RESULTS

### Neomycin increases plaque size of CVB3-Nancy and reovirus but not poliovirus.

**FIG 1:**
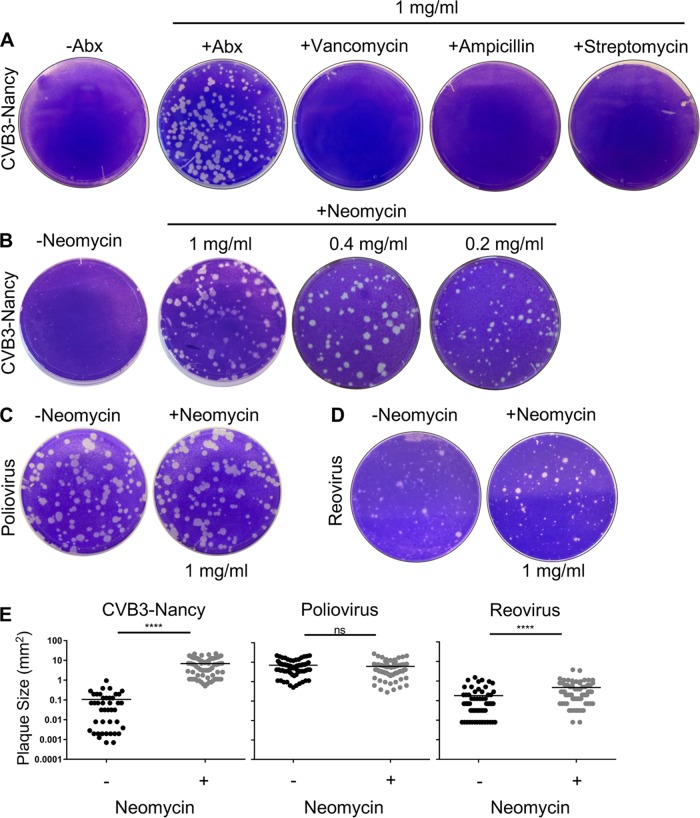
Effect of neomycin on plaque formation of CVB3-Nancy, poliovirus, and reovirus. (A) Effects of antibiotics on CVB3-Nancy plaque formation. HeLa cells were pretreated with or without 1 mg/ml of the indicated antibiotics prior to plating of 100 PFU of CVB3-Nancy on cells with agar overlays with or without 1 mg/ml of each antibiotic. Plates were stained with crystal violet. Abx, ampicillin, neomycin, streptomycin, and vancomycin mixture. (B) Effect of neomycin on CVB3-Nancy plaque formation. The experiment was performed as for panel A, in the presence or absence of neomycin at various concentrations. (C and D) Effects of neomycin on poliovirus (C) or type 3 Dearing reovirus (D). (E) Plaque size quantification of CVB3-Nancy, poliovirus, and reovirus in the presence or absence of 1 mg/ml of neomycin. Each symbol represents a plaque. ****, *P* < 0.0001 (unpaired two-tailed Student *t* tests). ns, not significant. All images are representative of results from at least 2 or 3 independent experiments.

To examine the effect of antibiotics on plaque formation of strain Nancy of coxsackievirus B3 (CVB3-Nancy), we infected a monolayer of HeLa cells that had been pretreated or not with 1 mg/ml of an antibiotic cocktail consisting of vancomycin, ampicillin, neomycin, and streptomycin. Following adsorption for 30 min, the inoculum was removed, and an agar overlay with or without antibiotics was added. To visualize plaques, plates were stained with crystal violet 2 days postinfection (dpi). When cells were exposed to the antibiotic cocktail, we observed a significant increase in CVB3-Nancy plaque size (Fig. 1A). Treatment with vancomycin, ampicillin, or streptomycin alone did not confer the large-plaque phenotype (Fig. 1A), but treatment with neomycin was sufficient for the large plaque phenotype (Fig. 1B). Lower concentrations of neomycin were also sufficient for large-plaque formation (Fig. 1B). We next determined whether neomycin also affects the plaque size of a closely related enteric virus, poliovirus, or an unrelated enteric virus, reovirus. When cells were pretreated or not with neomycin and infected with poliovirus, plaques were relatively large and no increase in plaque size was observed with neomycin treatment (Fig. 1C). However, neomycin treatment increased plaque size of type 3 Dearing reovirus, a double-stranded RNA virus (Fig. 1D). We quantified plaque size and found that when cells were exposed to neomycin, CVB3-Nancy plaques were 63-fold larger than in untreated cells and reovirus plaques were 2.6-fold larger than in untreated cells, but there was no significant effect on poliovirus plaque size (Fig. 1E). Overall, these data indicate that treatment with neomycin is capable of increasing plaque size CVB3-Nancy and reovirus but not poliovirus.

### Effect of neomycin on plaque sizes of different CVB3 strains.

**FIG 2:**
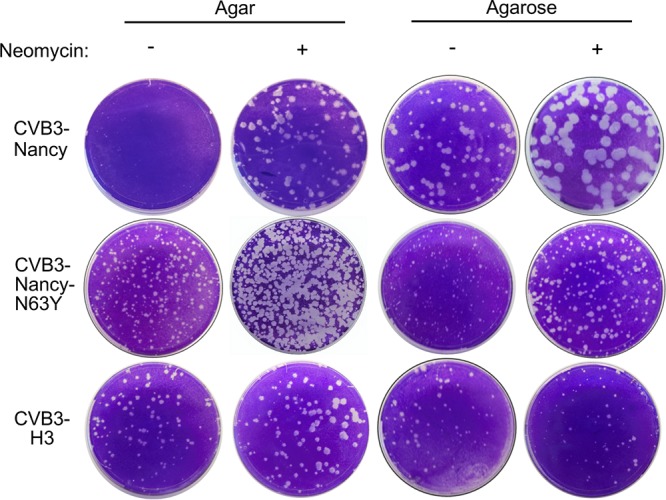
Effect of neomycin on plaque formation of different CVB3 strains. HeLa cells were pretreated or not with neomycin and were infected with approximately 100 PFU of CVB3-Nancy, CVB3-Nancy-N63Y, or CVB3-H3, and then agar or agarose overlays, containing or lacking 1 mg/ml of neomycin, were added. All images are representative of results from at least two independent experiments.

Since neomycin was capable of increasing the plaque size of CVB3-Nancy, we hypothesized that neomycin may also increase the plaque sizes of closely related CVB3 strains. To investigate this hypothesis, we first used CVB3-Nancy-N63Y, which is a CVB3-Nancy derivative that contains a single point mutation in the VP3 capsid protein, N63Y, which induces formation of large plaques in agar overlays due to reduced binding to sulfated glycans (17). We found that neomycin was also capable of increasing the plaque size of CVB3-Nancy-N63Y in agar overlays (Fig. 2), although the effect was less pronounced than with CVB3-Nancy due to the larger plaques of CVB3-Nancy-N63Y in untreated cells. We next examined if neomycin treatment could increase the plaque size of CVB3-H3, a strain of CVB3 that is more virulent in mice (18). Neomycin treatment did not alter CVB3-H3 plaque size (Fig. 2). These data indicate that in agar overlays, neomycin is capable of increasing the size of CVB3-Nancy and a closely related mutant, CVB3-Nancy-N63Y, but not CVB3-H3.

The small plaque size of CVB3-Nancy under agar overlays has been attributed to its binding to sulfated glycans present in agar, which limits viral diffusion (17). However, in agarose overlays, which contain low levels of sulfated glycans, CVB3-Nancy plaque size is significantly larger. Therefore, we sought to examine whether neomycin treatment can increase the plaque size of CVB3-Nancy, CVB3-Nancy-N63Y, and CVB3-H3 in agarose overlays. We found that neomycin increased the plaque size of CVB3-Nancy in the presence of an agarose overlay (Fig. 2), although the effect was diminished due to larger CVB3-Nancy plaques in agarose overlays. Similarly, CVB3-Nancy-N63Y plaques were slightly larger in the presence of neomycin (Fig. 2). However, in agarose overlays, the plaque size of CVB3-H3 was unaffected by neomycin treatment (Fig. 2). Overall, these data suggest that in both agar and agarose overlays, neomycin can increase the plaque sizes of CVB3-Nancy and CVB3-Nancy-N63Y but not of CVB3-H3.

### Early stages of CVB3-Nancy infection are unaffected by neomycin.

**FIG 3:**
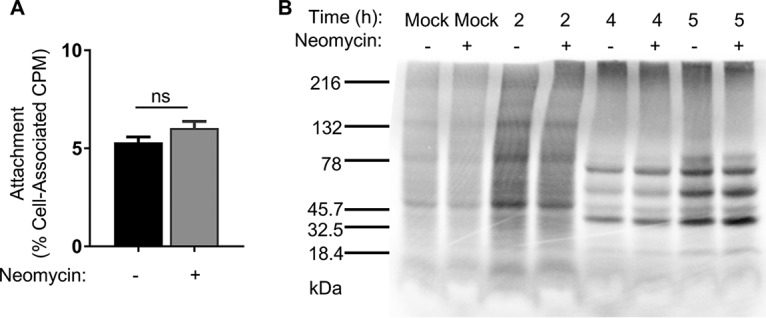
Effects of neomycin on early stages of the CVB3-Nancy replication cycle. (A) Cell attachment assay. A total of 1 × 10^6^ HeLa cells were pretreated or not with neomycin prior to incubation with 6,000 cpm (6 × 10^6^ PFU) of ^35^S-labeled CVB3-Nancy or no virus (mock) at 4°C for 20 min to promote viral binding. Cells were washed and ^35^S was quantified in a scintillation counter. Data are means ± SEMs (unpaired two-tailed Student *t* tests; *n* = 5 total data points from two independent experiments). (B) Viral protein synthesis assay. A total of 2.5 × 10^6^ HeLa cells that had been pretreated or not with neomycin were inoculated with CVB3-Nancy at an MOI of 20 for 30 min at 37°C. At the indicated time points, cells were washed and incubated in 1 ml of DMEM lacking methionine and cysteine supplemented with [^35^S]l-methionine and [^35^S]l-cysteine for 15 min at 37°C. Cell lysates from equal cell numbers were analyzed on an SDS-PAGE gel. Radiolabeled proteins were visualized using a phosphorlmager. The image is representative of results from three independent experiments.

Given that CVB3-Nancy generates large plaques in the presence of neomycin, we hypothesized that neomycin enhances viral replication. To test this hypothesis, we first determined whether early steps of the viral replication cycle are affected by neomycin. To examine viral attachment, we quantified binding of radiolabeled ^35^S-labeled CVB3-Nancy to HeLa cells in the presence or absence of neomycin pretreatment. HeLa cells were pretreated or not with neomycin overnight, followed by incubation with ^35^S-labeled virus at 4°C for 20 min. After washing, cell-associated ^35^S was quantified. ^35^S counts were the same for cells treated or not with neomycin, suggesting that neomycin does not affect viral attachment (Fig. 3A). We next sought to determine if viral translation is affected by neomycin treatment. Picornaviruses, including CVB3, initiate translation early in the viral life cycle, which results in shutoff of host protein synthesis (19, 20). HeLa cells that were pretreated or not overnight with neomycin were infected with CVB3-Nancy, and then at various time points cells were exposed to media containing ^35^S-labeled cysteine and methionine to label nascent proteins. Cell lysates were run on an SDS-PAGE gel, and ^35^S labeled proteins were imaged via a phosphorimager. We found that the amounts of labeled viral and cellular proteins were the same for cells treated with or without neomycin (Fig. 3B). Overall, these data suggest that neomycin does not affect the early stages of the CVB3-Nancy life cycle.

### Replication kinetics of CVB3-Nancy are unaffected by neomycin treatment.

**FIG 4:**
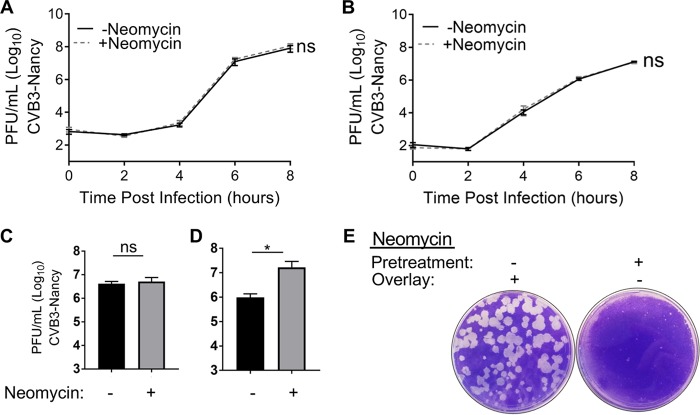
Effects of neomycin on CVB3-Nancy replication kinetics. Shown is the growth curve in the presence of liquid media (A) or agar overlay (B). Briefly, 1 × 10^6^ HeLa cells that had been pretreated or not with neomycin were inoculated with CVB3-Nancy at an MOI of 0.01. Virus was incubated for 30 min at 37°C, and either DMEM liquid (A) or 1% agar–1% DMEM mixture (B) with or without neomycin was added. At the indicated time points, intracellular virus was harvested and quantified by plaque assay. For panels A and B, *n* = 4 or 5 total data points from two independent experiments. To examine multicycle replication and spread, cells were infected with 100 PFU of CVB3-Nancy and either DMEM (C) or 1% agar–1% DMEM mixture (D) with or without neomycin, followed by plaque assay of cell-associated virus. For panels C and D, *n* = 6 total data points from three independent experiments. (E) HeLa cells were pretreated or not with 1 mg/ml of neomycin prior to plating of 100 PFU of CVB3-Nancy on cells with agar overlays with or without 1 mg/ml of neomycin. Plates were stained with crystal violet 48 hpi. *, *P* < 0.05 (unpaired two-tailed Student *t* tests). Images are representative of results from two independent experiments.

To examine whether neomycin affects the replication kinetics of CVB3-Nancy, we used single-cycle growth curve assays. HeLa cells that were pretreated or not with neomycin were infected with CVB3-Nancy at a multiplicity of infection (MOI) of 0.01, and cell-associated viral titers were determined over time. CVB3-Nancy titers were the same for cells treated and not treated with neomycin at each time point analyzed (Fig. 4A). We next determined whether the presence of an agar overlay could alter the replication kinetics of CVB3-Nancy, and whether the presence of neomycin in the agar overlay could alter CVB3-Nancy growth. For these experiments, HeLa cells were pretreated or not with neomycin and were infected for 30 min with CVB3-Nancy at an MOI of 0.01, followed by addition of an agar overlay with or without neomycin. At 0, 2, 4, 6, or 8 h postinfection (hpi), agar overlays were removed, cells were harvested, and cell-associated virus titers were determined by plaque assay on naive cells. We found no difference in CVB3-Nancy replication in the presence of an agar overlay, with or without neomycin (Fig. 4B). Given that neomycin did not affect single-cycle replication kinetics of CVB3-Nancy in either liquid media or agar overlay, we next determined whether neomycin could alter spread during multiple replication cycles. One million HeLa cells were inoculated with 100 PFU of CVB3-Nancy in the presence or absence of neomycin, and the cells were incubated for 24 h to allow multiple replication cycles to occur. Viral titers in the presence of liquid media showed no differences in viral yield with or without neomycin (Fig. 4C). However, when CVB3-Nancy was grown for 24 h in the presence of an agar overlay, a significant increase in viral yield was detected in the presence of neomycin (Fig. 4D). Overall these data indicate that a single cycle of replication of CVB3-Nancy was unaffected by neomycin treatment, but titers from multiple cycles were increased by neomycin treatment when agar overlays were present, suggesting that neomycin may aid viral spread in the presence of agar.

### Positive charge of neomycin contributes to generation of large CVB3-Nancy plaques.

Because neomycin increased the plaque size of CVB3-Nancy in agar overlays and neomycin increased the 24-h titers of CVB3-Nancy when an agar overlay was present, we hypothesized that neomycin enhances viral diffusion by overcoming inhibition by negatively charged compounds in agar overlays. Additionally, we hypothesized that neomycin must be present in the agar overlay to enhance plaque formation and that pretreatment of cells with neomycin would not be sufficient to increase plaque size when neomycin was not present in agar overlays. To test this, we pretreated cells with neomycin or left them untreated, infected them with 100 PFU of CVB3-Nancy, and added an agar overlay with or without neomycin. We found that large plaques formed in cells with neomycin in agar overlays regardless of whether cells had been pretreated with neomycin before infection. Conversely, small plaques formed in cells without neomycin in agar overlays regardless of whether they had been pretreated with neomycin (Fig. 4E). Thus, the presence of neomycin in the overlay is sufficient to increase the plaque size of CVB3-Nancy.

**FIG 5:**
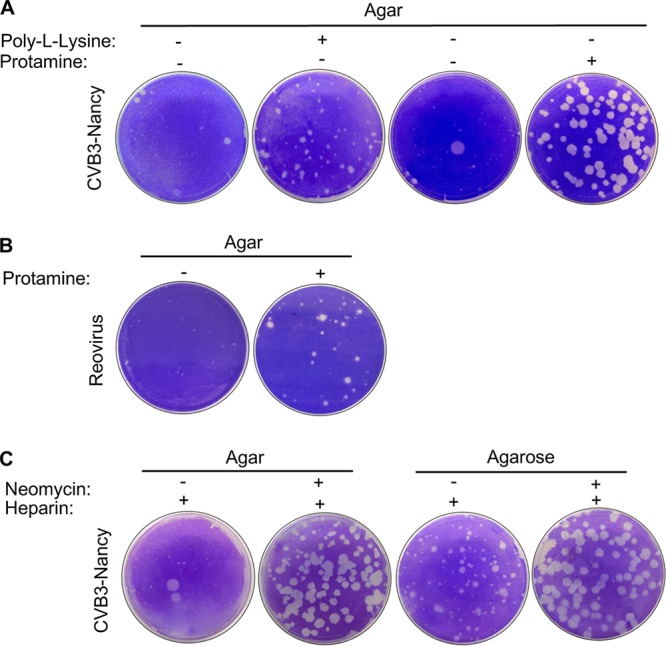
Effect of positively or negatively charged compounds on CVB3-Nancy and reovirus plaque formation. (A) A total of 8.8 × 10^6^ HeLa cells were infected with 100 PFU of CVB3-Nancy and agar overlays were added with or without 0.1 µM poly-l-lysine or 0.8 mg/ml of protamine (positively charged compounds). (B) A total of 1 × 10^6^ L929 cells were infected with 100 PFU of type 3 Dearing reovirus and agar overlays were added with or without 0.8 mg/ml of protamine. (C) A total of 8.8 × 10^6^ HeLa cells were infected with 100 PFU of CVB3-Nancy and neomycin-containing agar or agarose overlays were added with or without 1 mg/ml of heparin (negatively charged compound). Images are representative of results from two independent experiments.

Agar is rich in anionic sulfated polysaccharides, which inhibit some viruses by binding and preventing cell adsorption or diffusion (21–23), and cationic compounds can overcome this negative-charge inhibition of the agar overlay (22). Given that neomycin is also a positively charged compound (24), we hypothesized that neomycin increases CVB3-Nancy plaque size by overcoming the inhibitory negative charge of the agar overlay. To test this, we evaluated whether other positively charged compounds could also increase the plaque size of CVB3-Nancy. We found that two positively charged compounds, poly-l-lysine and protamine, increased the plaque size of CVB3-Nancy (Fig. 5A). Additionally, we found that protamine increased the plaque size of type 3 Dearing reovirus (Fig. 5B). We next determined whether neomycin was capable of overcoming the inhibitory effect of negatively charged heparin (17). We found that CVB3-Nancy was able to generate large plaques in the presence of heparin when neomycin was present (Fig. 5C). These data indicate that the positive charge of neomycin contributes to large-plaque formation of CVB3-Nancy and reovirus.

**FIG 6:**
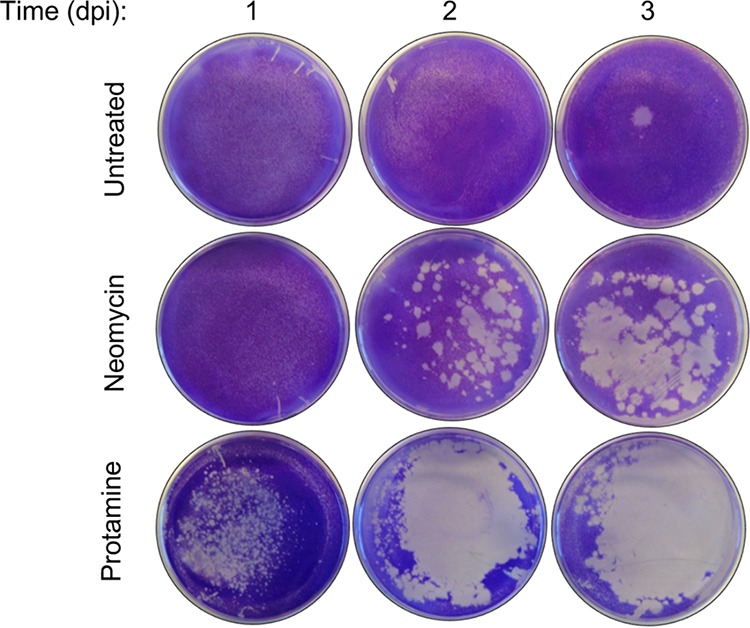
Effects of positively charged compounds on CVB3-Nancy diffusion. A total of 1 × 10^6^ HeLa cells in 60-mm tissue culture plates were overlaid with 4 ml of 1% agar–1% DMEM mixture that contained or lacked 1 mg/ml of neomycin or 0.8 mg/ml of protamine. Once the overlay solidified, 5 × 10^4^ PFU of CVB3-Nancy in 200 µl was added dropwise to the top of the overlay. Cells were placed at 37°C to allow diffusion of the virus through the overlay to the cell monolayer, and plates were stained with crystal violet at 1, 2, or 3 days postinfection (dpi) to reveal the extent of cell death from viral replication. Images are representative of results from two independent experiments.

Because other positively charged compounds phenocopy neomycin’s effects, we hypothesized that neomycin facilitates large-plaque formation of CVB3-Nancy by neutralizing negatively charged inhibitory molecules in agar, allowing the virus to diffuse more efficiently. To test this, we used a diffusion assay previously described by Wallis and Melnick (22). Agar overlays with or without neomycin or protamine were added to untreated and uninfected HeLa cells and allowed to solidify. Then 5 × 10^4^ PFU of CVB3-Nancy was added dropwise on top of the agar overlay. Virus was allowed to diffuse downward through the ∼1-cm-thick agar overlay and infect cells, and plates were stained with crystal violet at 1, 2, or 3 dpi to examine cell death. We found that CVB3-Nancy diffusion and subsequent cell death were greatly enhanced when either neomycin or protamine was added to the agar overlays, compared to the case with agar overlays with no treatment (Fig. 6). Overall, these results suggest that the positive charge of neomycin enhances CVB3-Nancy diffusion by overcoming the negative charge of agar overlays.

## DISCUSSION

Although it is known that antibiotics deplete the gut microbiota of mice and microbiota depletion reduces infection of certain enteric viruses, it remains unclear if antibiotics can directly affect enteric viruses independently of effects on the microbiota. Here we show that an aminoglycoside antibiotic, neomycin, increases the plaque size of CVB3-Nancy. In this study, we found that neomycin enhances CVB3-Nancy plaque formation through increased viral diffusion due to its positive charge.

CVB3-Nancy is a cell culture-adapted virus that has increased binding to heparan sulfate, a negatively charged sulfated polysaccharide that is present on the surface of cells and in agar overlays (17, 21). The presence of sulfated polysaccharides in agar overlays limits diffusion of certain heparan sulfate-binding viruses, which results in formation of small plaques (21). Interestingly, CVB3-Nancy-N63Y, a derivative of CVB3-Nancy that contains a single mutation that decreases virion binding to sulfated polysaccharides, also had increased plaque size due to neomycin treatment. CVB3-Nancy-N63Y has larger plaques in agar overlays, suggesting that its diffusion is less limited by the negative charge of the sulfated polysaccharides (Fig. 2). CVB3-H3 is a less culture-adapted and more pathogenic strain of CVB3, and CVB3-H3 formed large plaques in the presence or absence of neomycin (Fig. 2). Since neomycin had an effect on plaque formation only of CVB3-Nancy strains and reovirus, and not CVB3-H3 or poliovirus, neomycin may enhance diffusion only of viruses that bind to negatively charged sulfated polysaccharides.

Anionic polymers, such as sulfated polysaccharides present in agar overlays, have inhibitory effects by limiting adsorption of newly formed virions to cells (22, 25, 26). Protamine, a positively charged compound, enhances the plaque sizes of encephalomyocarditis virus and adenovirus (22, 25, 26), promotes diffusion of enterovirus (27), and enhances infectivity of rabies virus (28). In agreement with previous studies, we found that protamine enhanced plaque size of CVB3-Nancy and reovirus in the presence of an agar overlay (Fig. 5). We also found that poly-l-lysine, a compound with a charge to similar to that of neomycin (29), also increased CVB3-Nancy plaque size in the presence of an agar overlay. To confirm that the positive charge of neomycin enhances viral diffusion, we performed a diffusion assay and found that neomycin- or protamine-treated overlays enhanced CVB3-Nancy diffusion.

In conclusion, we found that positively charged compounds, such as neomycin, poly-l-lysine, and protamine, aid CVB3-Nancy and CVB3-N63Y diffusion in the presence of either an agar or agarose overlay. This work provides insight into methods to enhance plaque formation and reveals that a commonly used antibiotic can have microbiota-independent effects on a virus.

## MATERIALS AND METHODS

### Cells and virus.

HeLa cells were grown in Dulbecco’s modified Eagle’s medium (DMEM) supplemented with 10% calf serum (Sigma-Aldrich) and 1% penicillin-streptomycin (Sigma-Aldrich). The CVB3-Nancy and CVB3-H3 infectious clones were obtained from Marco Vignuzzi (Pasteur Institute, Paris, France), and the CVB3-Nancy-N63Y infectious clone was previously generated from CVB3-Nancy by site-directed mutagenesis (17). The poliovirus infectious clone was serotype 1 Mahoney (30). Viral stocks were prepared as previously described (17), and viral titers were determined by plaque assay as previous described (17, 31). Briefly, monolayers of HeLa cells (CVB3 or poliovirus) were infected for 30 min, followed by addition of an overlay containing media and 1% agar (Becton, Dickinson) or 1% SeaKem LE agarose (Lonza). Following incubation, plaques were visualized by staining with an alcoholic solution of crystal violet. Type 3 Dearing reovirus was generated in L929 cells and gradient purified as previously described (32). L929 cells were grown in DMEM supplemented with 5% fetal bovine serum (Sigma-Aldrich) and 1% penicillin-streptomycin (Sigma-Aldrich). Reovirus plaque assays were performed as described previously using 1 × 10^6^ L929 cells seeded in 6-well plates using an overlay containing media and 1% agar (Becton, Dickinson) (32). Following incubation for 7 days, plaques were visualized by staining with an alcoholic solution of crystal violet.

### CVB3 cell attachment.

^35^S-labeled CVB3-Nancy was generated as previously described (8, 17). Briefly, cells were infected with CVB3-Nancy in the presence of [^35^S]l-methionine and [^35^S]l-cysteine express labeling mix (PerkinElmer), and viruses in cell lysates were purified using CsCl gradient ultracentrifugation (8, 17). A total of 6,000 cpm (6 × 10^6^ PFU) was incubated with 1 × 10^6^ HeLa cells that had been pretreated or not with 1 mg/ml of neomycin (Research Products International) at 4°C for 20 min to promote viral binding. Cells were washed three times with ice-cold phosphate-buffered saline (PBS) to remove unbound labeled virus and were trypsinized, and ^35^S was quantified in a scintillation counter (Beckman Coulter; LS6500 multipurpose scintillation counter).

### CVB3 translation assay.

A total of 2.5 × 10^6^ HeLa cells were pretreated or not with 1 mg/ml of neomycin for 16 h and were inoculated with CVB3-Nancy at an MOI of 20 for 30 min at 37°C. Inoculum was aspirated, cells were washed with PBS, and complete DMEM was added. At 2, 4, and 5 h postinfection (hpi), cells were washed and incubated in 1 ml of DMEM lacking methionine and cysteine (Sigma-Aldrich) with 55 µCi of ^35^S express labeling mix (PerkinElmer) for 15 min at 37°C. Cells were harvested and lysed in buffer containing 10 mM Tris (pH 8), 10 mM NaCl, 1.5 mM MgCl_2_, and 1% NP-40, and nuclei were removed by centrifugation. Supernatants from equal cell numbers were analyzed on 4 to 20% Mini-PROTEAN TGX precast protein gels (Bio-Rad). Gels were dried at 80°C for 1 h and exposed to a phosphorimager screen overnight. Radiolabeled proteins were visualized using a phosphorlmager (Typhoon FLA 9500).

### Viral growth curves.

A total of 1 × 10^6^ HeLa cells pretreated overnight or not with 1 mg/ml of neomycin were inoculated with CVB3-Nancy at an MOI of 0.01. Virus was incubated for 30 min at 37°C to promote viral binding, inoculum was aspirated, the cell monolayer was washed once in PBS, and 2 ml of either DMEM or 1% agar–1% DMEM mixture with or without 1 mg/ml of neomycin was added. At 0, 2, 4, 6, and 8 h postinfection, medium was removed, the cell monolayer was washed once in PBS, and cells were trypsinized and pelleted. Intracellular virus was harvested by freeze-thawing three times. For 24-hpi assays, cells were infected with 100 PFU of CVB3-Nancy and 2 ml of either DMEM or 1% agar–1% DMEM mixture with or without 1 mg/ml of neomycin was added. Plaque assay, as described above, was used to quantify amount of intracellular PFU.

### Viral diffusion assay.

A total of 1 × 10^6^ uninoculated HeLa cells in 60-mm tissue culture plates were overlaid with 4 ml of 1% agar–1% DMEM mixture with or without 1 mg/ml of neomycin or 0.8 mg/ml of protamine. Once the overlay had solidified, 5 × 10^4^ PFU of CVB3-Nancy in 200 µl was added dropwise to the overlay. Cells were placed at 37°C to allow diffusion of the virus through the overlay to the cell monolayer. Plates were stained with crystal violet at 1, 2, or 3 days postinfection.

### Statistical analysis.

The difference between groups were examined by the unpaired two-tailed Student *t* test. Error bars in figures represent the standard errors of the means. A *P* value of <0.05 was considered significant. All analyses of data were performed using GraphPad Prism version 7.00 for Windows, GraphPad Software, La Jolla, CA.
